# Prospective Study of Isolated Recurrent Tumor Re-irradiation With Carbon-Ion Beams

**DOI:** 10.3389/fonc.2019.00181

**Published:** 2019-03-27

**Authors:** Katsuyuki Shirai, Tatsuya Ohno, Jun-ichi Saitoh, Masahiko Okamoto, Hiroyuki Katoh, Kazutoshi Murata, Hidemasa Kawamura, Atsushi Musha, Takanori Abe, Tatsuji Mizukami, Keiko Akahane, Takashi Nakano

**Affiliations:** ^1^Department of Radiology, Saitama Medical Center, Jichi Medical University, Omiya-ku, Japan; ^2^Gunma University Heavy Ion Medical Center, Maebashi, Japan

**Keywords:** carbon-ion radiotherapy, re-irradiation, prospective study, Bragg peak, isolated recurrent tumor

## Abstract

**Purpose:** To perform a prospective study to evaluate the efficacy and safety of isolated recurrent tumor re-irradiation with carbon-ion radiotherapy (RT).

**Methods and Materials:** The inclusion criteria were clinically proven recurrent tumors, measurable by computed tomography or magnetic resonance imaging, patients ≥ 16 years old, performance status scores between 0 and 2, isolated tumor at a previously irradiated site, and a life expectancy > 6 months. The exclusion criteria were tumor invasion into the gastrointestinal tract or a major blood vessel, uncontrolled infection, early recurrence (<3 months), and severe concomitant diseases. The primary end-point was the local control rate, the secondary end-points including the overall survival rate, and adverse events.

**Results:** Between December 2013 and March 2016, 22 patients were enrolled in this prospective study. All patients were re-irradiated with carbon-ion RT with radical intent. Five patients had rectal cancer, 4 had sarcoma, 4 had lung cancer, 3 had hepatic cell carcinoma, and 6 had other tumors. The median follow-up time was 26 months. Eight patients developed local recurrence, and the 1- and 2-year local control rates were 71 and 60%, respectively. Eight patients died of their cancers and 2 died of other diseases. The 1- and 2-year overall survival rates were 76 and 67%, respectively. There were no grade 2 or higher acute adverse events and 4 patients (18%) developed grade 3 late adverse events. The group with the longer interval (>16 months) between the first RT and re-irradiation had significantly better outcomes than the shorter interval group (≤ 16 months).

**Conclusions:** Re-irradiation, using carbon-ion RT with radical intent, had favorable local control and overall survival rates without severe toxicities for selected patients. Re-irradiation has the potential to improve clinical outcomes for isolated, local, recurrent tumors; further investigations are required to confirm the therapeutic efficacy.

## Introduction

Radiotherapy (RT) is widely performed for several types of tumors and is considered to be a curative and non-invasive treatment. Although the recent development of high precision RT has improved tumor control, local recurrence frequently occurs in advanced cases. Local tumor recurrence after RT is considered a refractory disease, and curative treatment options are limited. Salvage surgery is the mainstay for a curative treatment approach for these patients; however, comorbidity (e.g., bleeding and ruptured sutures) levels are high because the radiated tissues are fragile (1, 2). Repeating RT at the same site, i.e., re-irradiation, is difficult, because locally recurring tumors are more radio-resistant than original tumors and re-irradiation exceeds the tolerable dose of radiation for the surrounding normal tissue. Therefore, re-irradiation with curative intent is challenging. In fact, to date, previous re-irradiation was mostly performed as a palliative treatment (3, 4).

Carbon-ions have good dose-localizing properties, because of the Bragg peak, and the dose to the surrounding normal tissue can be minimized. Moreover, a carbon-ion beam has high biological effectiveness, resulting in favorable clinical outcomes even for radio-resistant tumors (5). Recently, carbon-ion RT has been reported to show efficacy for several radio-resistant tumors, such as bone and soft tissue sarcoma (6). Therefore, carbon-ion RT is expected to overcome local tumor recurrence after RT without severe, adverse events. Here, we performed a prospective study to evaluate the efficacy and safety of re-irradiation by using carbon-ion RT for isolated recurrent tumors after RT.

## Methods and Materials

### Study Design

All patients with isolated recurrent tumors after the first RT were prospectively treated with carbon-ion RT at the Gunma University Heavy Ion Medical Center (UMIN000014513). The inclusion criteria were as follows: a clinically proven recurrent tumor, tumor measurable by computed tomography (CT) or magnetic resonance imaging (MRI), age ≥ 16 years, performance status scores between 0 and 2, an isolated tumor occurring at a previously irradiated site, and a life expectancy of more than 6 months. The exclusion criteria were as follows: tumor invasion into the gastrointestinal tract or a major blood vessel, uncontrolled infection, early (<3 months after the first RT) tumor recurrence, and severe concomitant diseases.

The pretreatment evaluations included a physical examination, CT, MRI, and 18-fluorodeoxyglucose-positron emission tomography (FDG-PET). Patients were seen every month for the first 6 months and every 3 months thereafter. MRI and/or CT assessments were performed alternately every 3 months, and FDG-PET was carried out annually. The primary end-point was the local control rate, and the secondary end-points included the overall survival rate and adverse events. Acute and late adverse events were evaluated according to the Common Terminology Criteria for Adverse Events, version 4.0. This study was reviewed and approved by the relevant institutional review board (No. 1108).

### Carbon-Ion Radiotherapy

All patients provided written informed consent before undergoing carbon-ion RT. The details of RT techniques and treatment planning have been reported previously (7, 8). The dose of carbon-ion RT was expressed as “Gy (relative biological effectiveness [RBE]).” RBE was set as 3.0 from previous experimental data (5). Re-irradiation was performed with radical intent, and the policy of dose-fractionation and dose-constraint was the same as that of the first carbon-ion RT. For example, hepatic cell carcinoma patients received C-ion RT with 52.8 Gy (RBE) to 60.0 Gy (RBE) in four fractions for usual cases and 60.0 Gy (RBE) in 12 fractions for close-to-gastrointestinal tract cases (9, 10). There were no patients treated with concomitant therapy including chemotherapy, targeting therapy, or immunotherapy.

### Statistical Analyses

The local control and overall survival rates were estimated by the Kaplan–Meier method. To compare subgroups, univariate analyses were performed using the log-rank test. Analyses were performed using SPSS software (version 25.0; SPSS Inc., Chicago, IL, USA) and a *P* < 0.05 was considered statistically significant.

## Results

### Patient and Tumor Characteristics

**Figure 1:**
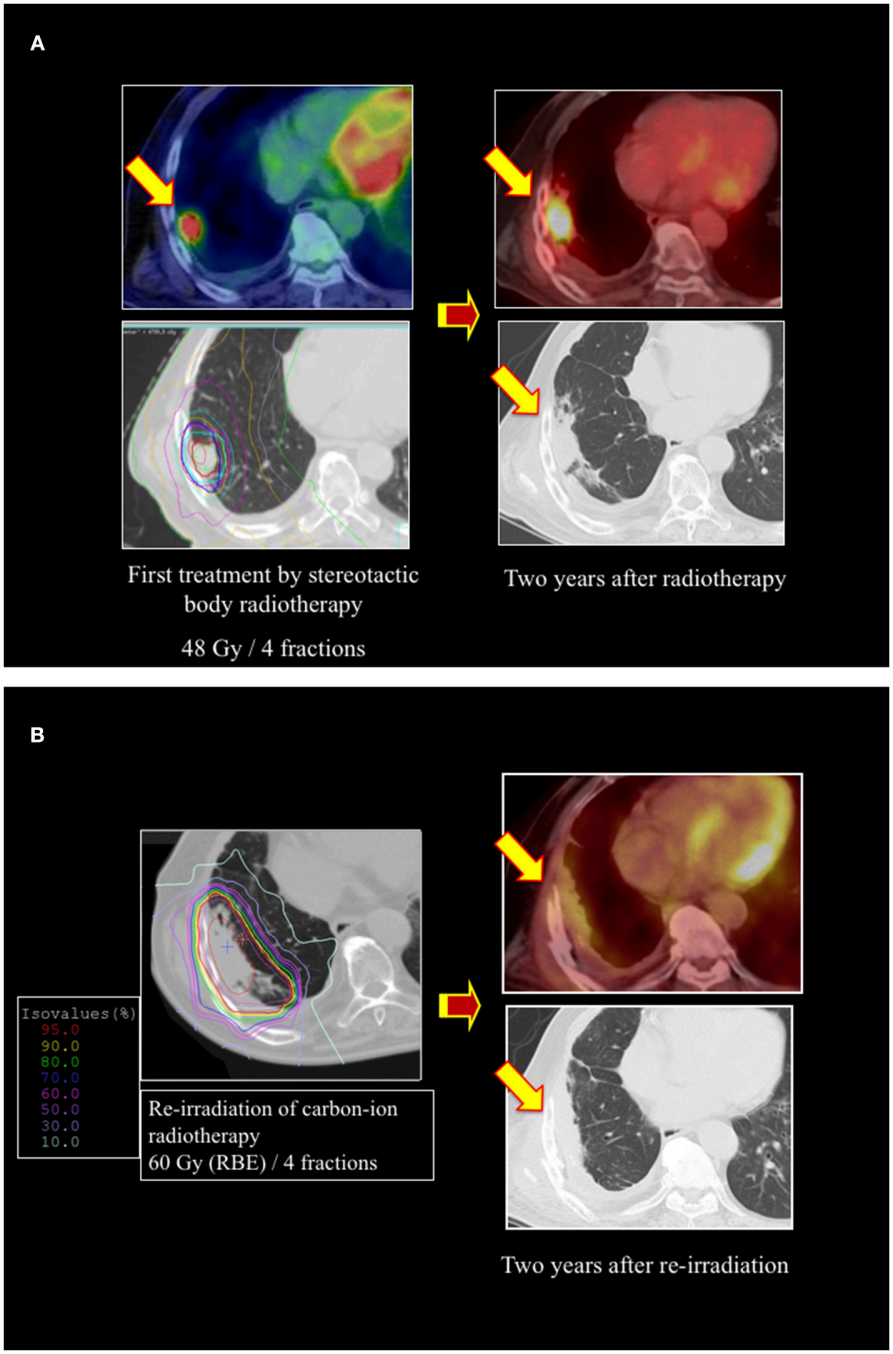
A representative case of re-irradiation by using carbon-ion radiotherapy (RT) for lung cancer. **(A)** Stereotactic body radiotherapy was performed for a 73-year-old male patient with stage I lung cancer as a first treatment. The local recurrence developed at the primary site, with surrounding fibrosis 2 years after the initial RT. **(B)** Re-irradiation was performed using carbon-ion RT of 60 Gy (relative biological effectiveness [RBE]) in 4 fractions. There was no local recurrence or metastasis 2 years after re-irradiation.

Between December 2013 and March 2016, 22 patients were enrolled in this prospective study (Supplemental Table 1). All patients were re-irradiated with radical intent. The median patient age at re-irradiation was 67 years (range: 17–89), and there were 13 male patients (59%). There were 14 patients with recurrence of primary tumors and 8 with recurrence in the lymph nodes. After the first RT, centrally and marginally located recurrence was observed in 20 and 3 patients, respectively. As a first treatment, photon therapy was performed in 9 patients and carbon-ion RT was performed in 13. There were 5 patients with rectal cancer, 4 with lung cancer, 4 with sarcoma, 3 with hepatic cell carcinoma, and 6 with other tumors. The median interval between the first RT and re-irradiation with carbon-ion RT was 16 months. A representative case of re-irradiation for lung cancer is shown in Figure 1.

### Clinical Outcomes

**Figure 2:**
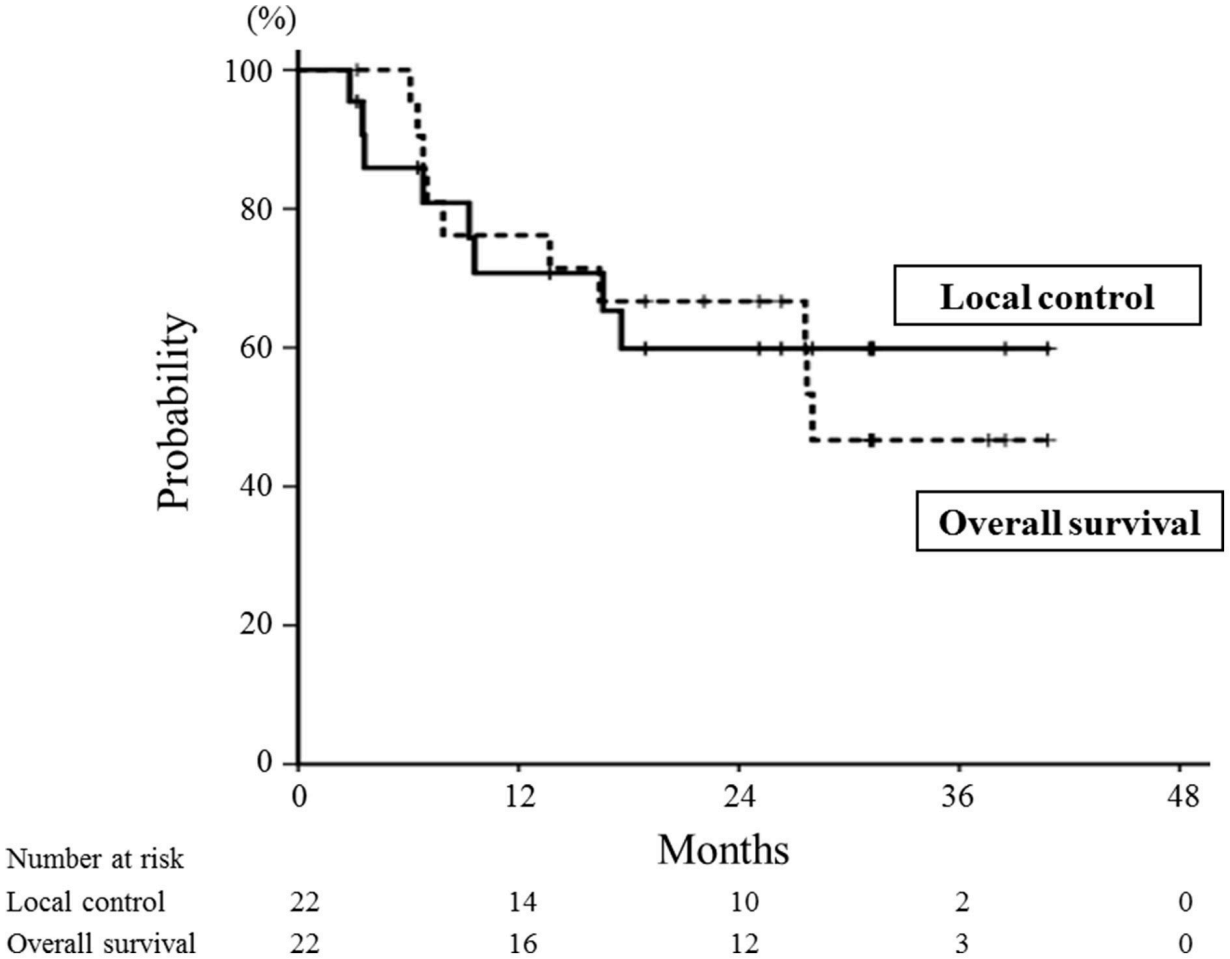
Local control and overall survival curves for all patients treated with re-irradiation by means of carbon-ion radiotherapy. The 1- and 2-year local control rates were 71 and 60%, respectively (solid line). The 1- and 2-year overall survival rates were 76 and 67%, respectively (dotted line).

**Figure 3:**
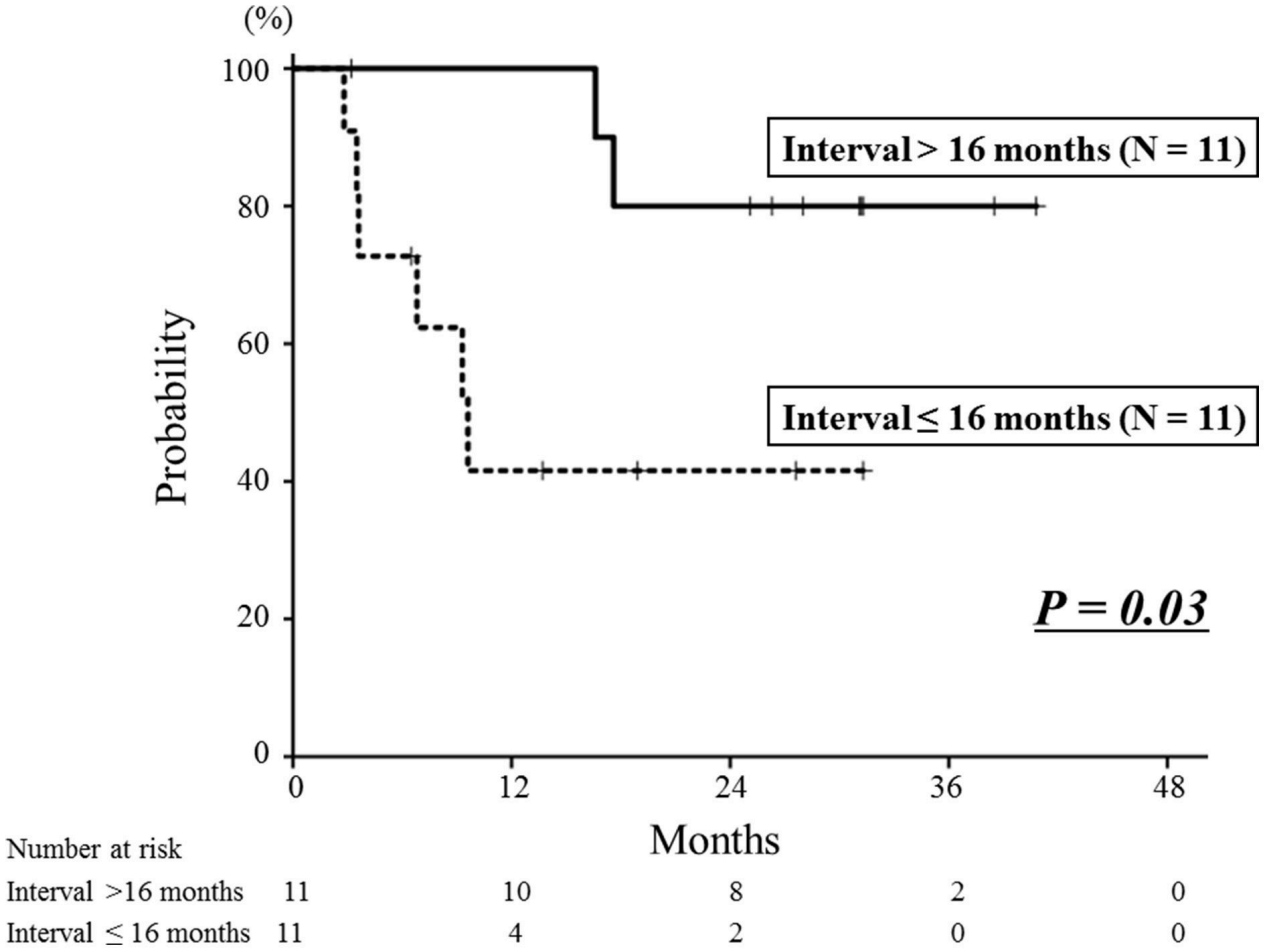
Local control curves according to the interval between the first radiotherapy session and re-irradiation. The 2-year local control rate of the longer interval group was 80%, which was significantly better than that of the shorter interval group of 42% (*P* = 0.03).

The median follow-up time after re-irradiation was 26 months (range: 3–41 months). The details of the first RT and re-irradiation with carbon-ion RT are shown in Supplemental Table 2. During the follow-up, 8 patients developed local recurrence, and the 1- and 2-year local control rates were 71 and 60%, respectively (Figure 2). The interval between the first RT and re-irradiation was a significant factor for local control (Figure 3, *P* = 0.03). The other patient and tumor characteristics were not associated with local control (Table 1).

**Table 1:** Univariate analysis for local control and overall survival.

**Characteristics**		***n* = 22**	**Local control**		**Overall survival**	
			**At 2-year follow-up**	***P*-value**	**At 2-year follow-up**	***P*-value**
Age (years)	≥ 67	11	64%		64%	
	< 67	11	53%	0.88	70%	0.67
Sex	Male	13	48%		62%	
	Female	9	75%	0.30	75%	0.93
Recurrent site	Primary tumor	14	52%		64%	
	Lymph node	8	71%	0.57	71%	0.84
First radiotherapy	Photon	9	50%		88%	
	Carbon-ion	13	68%	0.65	54%	0.35
Interval between the first radiotherapy and re-irradiation	> 16 months	11	80%		100%	
	≤16 months	11	42%	0.03	36%	< 0.01
Disease	Rectal cancer	5	50%		100%	
	Sarcoma	4	50%		25%	
	Lung cancer	4	50%		75%	
	Hepatic cell carcinoma	3	100%		67%	
	Other tumors	6	67%	0.76	67%	0.67

**Figure 4:**
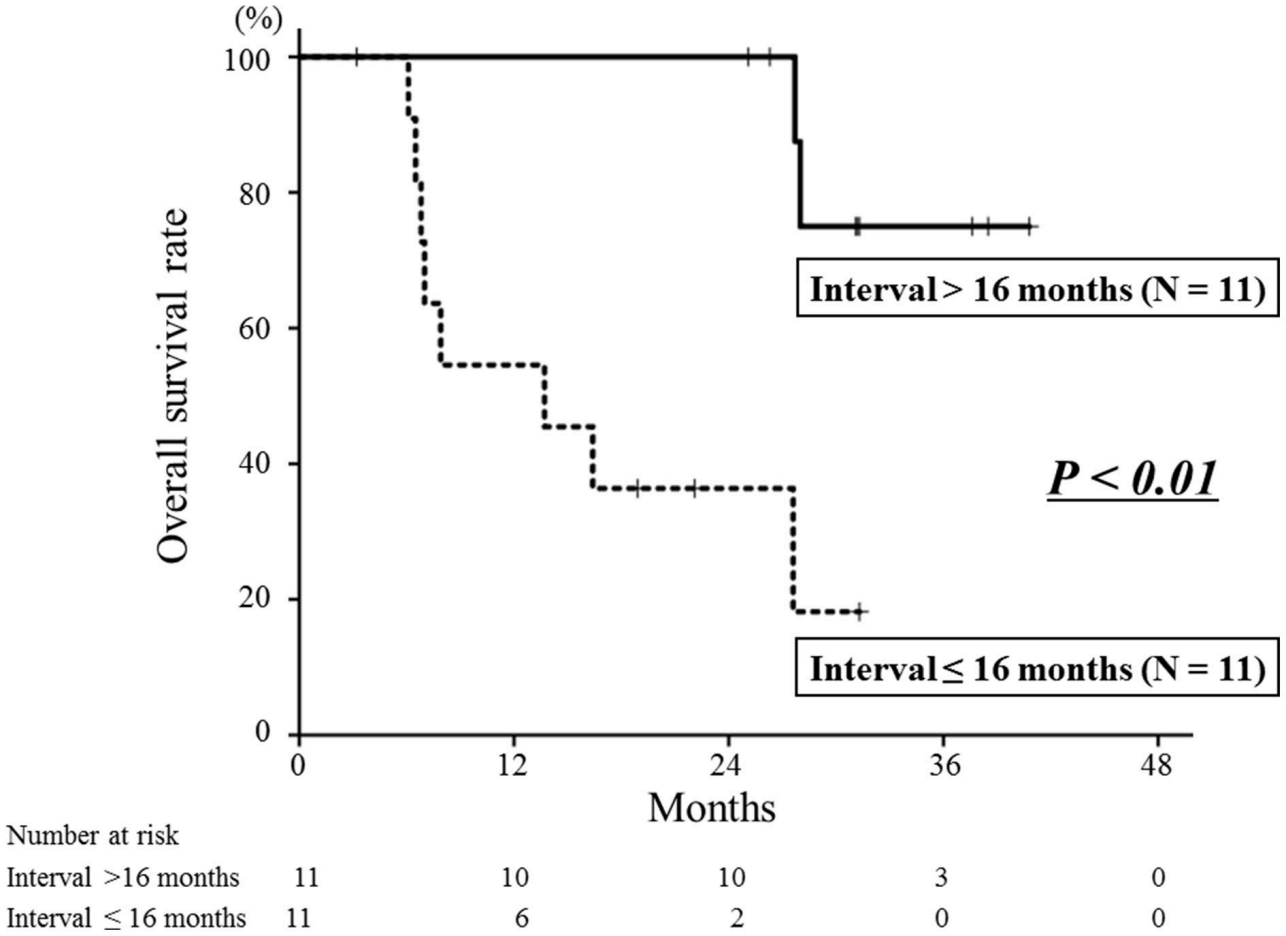
Overall survival curves according to the interval between the first radiotherapy and re-irradiation. The 2-year overall survival rate of the longer interval group was 100%, which was significantly better than that of the shorter interval group of 36% (*P* < 0.01).

Eight patients died of their cancers and 2 died of other diseases. The 1- and 2-year overall survival rates were 76 and 67%, respectively (Figure 2). The interval between the first RT and re-irradiation was a significant factor for overall survival (Figure 4, *P* < 0.01). The other patient and tumor characteristics were not associated with overall survival (Table 1).

### Adverse Events

Acute and late adverse events are shown in Supplemental Table 3. There were no grade 2 or higher acute adverse events. There were 4 patients (18%) with grade 3 late adverse events. Three patients had grade 3 urinary tract obstructions due to ureteral strictures, which were possibly associated with the effect of re-irradiation to pelvic tumors. All these patients were treated with ureteral stent insertions, and their symptoms immediately improved. One patient with sarcoma at the shoulder developed grade 3 peripheral motor neuropathy of the upper limb and a grade 3 skin ulcer, requiring plastic surgery. Although he was treated several times with photon therapy, carbon-ion RT, and surgery for the repeated local recurrence, this re-irradiation was considered one of the factors in the development of adverse events. There were no grade 4 or higher late adverse events.

## Discussion

We here report the clinical outcomes of a prospective study of re-irradiation using carbon-ion RT in 22 patients with isolated tumor recurrence after the first RT. The 2-year local control and overall survival rates were 60 and 67%, respectively. There were no grade 2 or higher acute adverse events. Although 4 patients (18%) developed grade 3 late adverse events, there were no grade 4 or higher events.

Recently, re-irradiation by means of stereotactic body radiotherapy and proton therapy have been reported for head and neck tumors, lung cancer, and abdominal tumors (Table 2). In head and neck tumors, the 2-year overall survival rates were 16–43% for photon and proton therapy (12–15). Grade 3 or higher late adverse events were observed in 13–21% of the patients and treatment-related death was observed in 2–8% for these patients. In particular, carotid blowout syndrome should be carefully considered as a life-threatening complication of head and neck tumors after re-irradiation. Jensen et al. reported a retrospective study of carbon-ion RT for head and neck tumors; these findings revealed that the 2-year overall survival rate was 63% and that there was no treatment-related death. In lung cancer, the 2-year overall survival rates were 18–74% in photon and proton therapy (17–20). Most studies have not shown local control, and 1 study of re-irradiation using proton therapy showed a 2-year local control rate of 24% (17). Severe radiation pneumonitis often developed, and grade 3 or higher late adverse events were observed in 9–30% of cases. In our study, there was no severe radiation pneumonitis after re-irradiation. A retrospective study of carbon-ion RT for lung cancer by Karube et al. showed that the 2-year overall survival rate was 67% without severe pneumonitis (16). In abdominal tumors, the 2-year overall survival rates were 30 to 37% for proton and photon therapy (23, 25). One prospective study of proton therapy for recurrent esophageal cancer found that grade 5 esophageal fistulas and ulcers were observed in 14% of patients during the acute and late periods (22). A retrospective study of photon therapy for hepatocellular carcinoma showed that severe RT-induced liver disease (grade 3 or higher) was observed in 36% and treatment-related death was 25% of the cases (24). Gastrointestinal and liver adverse events should be carefully assessed when re-irradiation is performed for abdominal tumors. The patients in our study exhibited no severe liver dysfunction or intestinal perforation. Moreover, Shiba et al. reported a retrospective study of carbon-ion RT for gynecologic cancer and found that the 3-year overall survival rate was 74% without intestinal toxicity (21).

**Table 2:** Previous studies of re-irradiation with carbon-ion, proton, and photon beams for various tumors.

**Reference**	**Disease type**	**Treatment**	**Study design**	**Patient number**	**Local control**	**Overall survival**	**Late grade ≥ 3**	**Late grade 5**
Jensen et al. (11)	Head and neck	Carbon-ion	Retrospective	52	47% (2 y)	63% (2 y)	15%	0%
McDonald et al. (12)	Head and neck	Proton	Retrospective	61	80% (2 y)	33% (2 y)	15%	5%
Romesser et al. (13)	Head and neck	Proton	Multi-institutional retrospective	92	55% (2 y)	43% (2 y)	13%	2%
Spencer et al. (14)	Head and neck	Photon	Prospective	81	-	16% (2 y)	-	7%
Yamazaki et al. (15)	Head and neck	Photon	Multi-institutional retrospective	107	64% (2 y)	35% (2 y)	21%	8%
Karube et al. (16)	Lung ca.	Carbon-ion	Retrospective	29	67% (2 y)	69% (2 y)	0%	0%
McAvoy et al. (17)	Lung ca.	Proton	Retrospective	33	24% (2 y)	33% (2 y)	30%	0%
Chao et al. (18)	Lung ca.	Proton	Multi-institutional prospective	57	-	43% (2 y)	12%	10%
Ebara et al. (19)	Lung ca.	Photon	Retrospective	44	-	18% (2 y)	9%	0%
Liu et al. (20)	Lung ca.	Photon	Retrospective	77	-	74% (2 y)	21%	1%
Shiba et al. (21)	Gynecologic ca.	Carbon-ion	Retrospective	16	94% (3 y)	74% (3 y)	0%	0%
Fernandes et al. (22)	Esophageal ca.	Proton	Prospective	14	-	14 months (MST)	29%	14% (acute and late)
Boimel et al. (23)	Pancreatic ca.	Proton	Retrospective	15	72% (1 y)	30% (2 y)	0%	0%
Huang et al. (24)	Hepatic cell carcinoma	Photon	Retrospective	36	-	-	36%	25%
Abusaris et al. (25)	Pelvic tumor	Photon	Retrospective	27	53% (2 y)	37% (2 y)	0%	0%
This study	Several diseases	Carbon-ion	Prospective	22	60% (2 y)	67% (2 y)	18%	0%

*Ca., cancer; y, year; MST, median survival time*.

It is difficult to compare carbon-ion RT with other forms of RT because these studies included several types of cancer (Table 2). However, our current and previously reported studies of carbon-ion RT showed 2- or 3-year overall survival rates of 63–74%, which were better outcomes than those obtained with proton and photon therapy (11, 16, 21). This may be because carbon-ion beams have a higher radiation biological efficacy than photon or proton therapies. Recent high precision therapy using photon and proton have also developed, and further investigations of re-irradiation are required to compare carbon-ion RT with other RT modalities.

Severe late adverse events were relatively uncommon (grade 3 or higher were 0–18%) and treatment-related death was not observed in carbon-ion RT studies; thus, this therapy seems safer than either photon or proton therapy. The reduction in adverse events with carbon-ion RT may be associated with its good dose-localizing properties, improving the focus of the radiation on the tumor. These results indicate that carbon-ion beams have strong potential for use as an optical beam for re-irradiation of local tumor recurrence after the initial RT.

In this study, tumor invasions into the gastrointestinal tract or a major blood vessel were excluded to avoid high irradiation of these serial organs. Therefore, intestinal perforations or arterial ruptures may not be observed with carbon-ion RT in the selected patients. Urinary tract obstruction due to a ureteral stricture was observed in 3 patients, indicating that the ureter should be considered as an organ at risk during re-irradiation. On the other hand, severe adverse events were uncommon in parallel organs, such as the lung and liver. Also, it may be relatively safe to re-irradiate previously irradiated tissue that has no function, such as fibrosis. Further study is required to establish the dose constraints in a re-irradiation setting.

It is important to identify optimal candidates for re-irradiation, because this treatment has a potential risk of severe adverse events. In this study, the interval between the first RT and re-irradiation was a significant factor for local control and overall survival. The longer interval group (≥16 months) had a better prognosis than the shorter interval group (< 16 months). In studies of re-irradiation for lung cancer, it has been reported that a longer interval between the first RT and re-irradiation was associated with better survival (26, 27). A multi-institutional cohort study of re-irradiation for head and neck tumors indicated that more than a 2-year interval from the first RT to re-irradiation is a factor indicating better prognosis (28). Also, slow-growing recurrent tumors can be less malignant than rapid growing tumors. These results indicate that recurrent tumors associated with longer periods after the first RT might be optimal candidates for re-irradiation with carbon-ion RT.

The present study had a few limitations, such as the small number of patients (*n* = 22) and the single-institutional study design. However, there have been few prospective studies of re-irradiation in general, and re-irradiation using carbon-ion RT has not been reported previously. Therefore, this study offers useful information on the effectiveness of carbon-ion as a re-irradiation therapy. Further, multi-institutional studies are required to further validate the efficacy of re-irradiation with carbon-ion RT.

In conclusion, re-irradiation with carbon-ion RT with radical intent offered favorable local control and overall survival rates, without severe toxicities, for the selected patients. Such re-irradiation has the potential to improve the clinical outcomes for isolated local recurrent tumors previously irradiated with photon or particle therapy. Further investigations are required to confirm its therapeutic efficacy in a multi-institutional setting.

## Data Availability

All datasets generated for this study are included in the manuscript and/or the Supplementary Files.

## Ethics Statement

This study was reviewed and approved by an institutional review board at Gunma University Heavy Ion Medical Center (No. 1108). The protocol was performed in accordance with the Declaration of Helsinki and all patients provided their written informed consent.

## Author Contributions

KS, TO, and JS designed and directed the analysis. KS, MO, HirK, KM, and HidK generated a database and performed data collection. KS, AM, TA, TM, and KA contributed to the analysis of the results and performed statistical analysis. TO and TN supervised the project.

### Conflict of Interest Statement

The authors declare that the research was conducted in the absence of any commercial or financial relationships that could be construed as a potential conflict of interest.
