# Supplementary material for: Prospective Study of Isolated Recurrent Tumor Re-irradiation With Carbon-Ion Beams
Source: Front Oncol. 2019 Mar 27;9:181. doi: 10.3389/fonc.2019.00181 (PMC6445888; doi:10.3389/fonc.2019.00181)
Supplement: Supplementary file 1 [file Data_Sheet_1.docx]

**Supplemental Table 1. Patient and tumor characteristics**

| Characteristics |  | N | Percentage |
| --- | --- | --- | --- |
| Age, median (years) |  | 67 | Range, 17–89 |
| Sex | Male | 13 | 59 |
|  | Female | 9 | 41 |
| Recurrent site | Primary tumor | 14 | 64 |
|  | Lymph node | 8 | 36 |
| First radiotherapy | Photon | 9 | 41 |
|  | Carbon-ion | 13 | 59 |
| Disease | Rectal cancer | 5 | 23 |
|  | Lung cancer | 4 | 18 |
|  | Sarcoma | 4 | 18 |
|  | Hepatic cell carcinoma | 3 | 14 |
|  | Cervical cancer | 2 | 9 |
|  | Sigmoid colon cancer | 1 | 5 |
|  | Anal canal cancer | 1 | 5 |
|  | Head and neck cancer | 1 | 5 |
|  | Esophageal cancer | 1 | 5 |

**Supplemental Table 2. Detail of the first radiotherapy and re-irradiation of carbon-ion radiotherapy**

| Disease | Patient | First RT | Recurrent disease type | Duration between the first RT and re-irradiation | Re-irradiation of carbon-ion radiotherapy | Clinical outcome | Follow-up time from re-irradiation |
| --- | --- | --- | --- | --- | --- | --- | --- |
| Lung cancer | Male, 73 y | Photon, 48.0 Gy / 4 fr | Primary tumor | 25.9 months | 60.0 Gy (RBE) / 4 fr | Alive | 31.1 months |
| Lung cancer | Male, 66 y | Photon, 60.0 Gy / 30 fr | Primary tumor | 9.7 months | 64.0 Gy (RBE) /16 fr | Alive | 22.1 months |
| Lung cancer | Male, 77 y | Carbon-ion, 60.0 Gy (RBE) / 4 fr | Primary tumor | 12.3 months | 60.0 Gy (RBE) / 4 fr | Dead | 6.8 months |
| Lung cancer | Male, 76 y | Carbon-ion, 52.8 Gy (RBE) / 4 fr | Primary tumor | 24.0 months | 60.0 Gy (RBE) / 4 fr | Alive | 31.2 months |
| Sarcoma | Male, 17 y | Carbon-ion, 70.4 Gy (RBE) / 16 fr | Primary tumor | 11.4 months | 57.6 Gy (RBE) / 12 fr | Dead | 13.7 months |
| Sarcoma | Male, 79 y | Carbon-ion, 70.4 Gy (RBE) / 16 fr | Primary tumor | 16.0 months | 57.6 Gy (RBE) / 12 fr | Dead | 7.0 months |
| Sarcoma | Male, 79 y | Carbon-ion, 67.2 Gy (RBE) / 16 fr | Primary tumor | 3.7 months | 67.2 Gy (RBE) / 16 fr | Alive | 31.3 months |
| Sarcoma | Female, 69 y | Carbon-ion, 67.2 Gy (RBE) / 16 fr | Primary tumor | 12.8 months | 64.0 Gy (RBE) / 16 fr | Dead | 16.4 months |
| Hepatic cell carcinoma | Female, 74 y | Carbon-ion, 52.8 Gy (RBE) / 4 fr | Primary tumor | 24.3 months | 60.0 Gy (RBE) / 4 fr | Dead | 28.0 months |
| Hepatic cell carcinoma | Male, 58 y | Carbon-ion, 52.8 Gy (RBE) / 4 fr | Primary tumor | 14.5 months | 60.0 Gy (RBE) / 12 fr | Dead | 6.5 months |
| Hepatic cell carcinoma | Male, 89 y | Carbon-ion, 52.8 Gy (RBE) / 4 fr | Primary tumor | 28.2 months | 60.0 Gy (RBE) / 4 fr | Alive | 31.3 months |
| Rectal cancer | Male, 47 y | Photon, 50.0 Gy / 25 fr | Primary tumor | 44.3 months | 73.6 Gy (RBE) / 16 fr | Dead | 27.7 months |
| Rectal cancer | Male, 65 y | Photon, 60.0 Gy / 30 fr | Primary tumor | 75.5 months | 64.0 Gy (RBE) / 16 fr | Alive | 37.6 months |
| Salivary cancer | Female, 62 y | Photon, 64.8 Gy / 36 fr | Primary tumor | 149.4 months | 57.6 Gy (RBE) / 16 fr | Alive | 38.5 months |
| Rectal cancer | Female, 54 y | Photon, 59.4 Gy / 33 fr | Lymph node | 25.1 months | 57.6 Gy (RBE) / 16 fr | Alive | 3.2 months |
| Sigmoid colon cancer | Female, 61 y | Photon, 50.0 Gy / 25 fr | Lymph node | 26.7 months | 52.8 Gy (RBE) / 12 fr | Alive | 40.8 months |
| Anal canal cancer | Female, 81 y | Photon, 50.0 Gy / 25 fr | Lymph node | 7.5 months | 64.0 Gy (RBE) / 16 fr | Dead | 27.6 months |
| Rectal cancer | Female, 69 y | Carbon-ion, 73.6 Gy (RBE) / 16 fr | Lymph node | 14.6 months | 57.6 Gy (RBE) / 12 fr | Alive | 18.9 months |
| Rectal cancer | Male, 54 y | Carbon-ion, 73.6 Gy (RBE) / 16 fr | Lymph node | 36.6 months | 57.6 Gy (RBE) / 12 fr | Alive | 25.1 months |
| Cervical cancer | Female, 32 y | Carbon-ion, 57.6 Gy (RBE) / 16 fr | Lymph node | 4.6 months | 57.6 Gy (RBE) / 12 fr | Dead | 7.9 months |
| Cervical cancer | Female, 66 y | Carbon-ion, 55.2 Gy (RBE) / 16 fr | Lymph node | 16.9 months | 57.6 Gy (RBE) / 12 fr | Alive | 26.3 months |
| Esophageal cancer | Male, 68 y | Photon, 56.0 Gy / 28 fr | Lymph node | 11.6 months | 52.8 Gy (RBE) / 12 fr | Dead | 6.1 months |

RT, radiotherapy; fr, fractions; RBE, relative biological effectiveness; y, years.

**Supplemental Table 3. Acute and late adverse events for all patients**

| Acute adverse events | Grade 2 | Grade 3 | Grade 4 |
| --- | --- | --- | --- |
| Mucositis | 0 (0%) | 0 (0%) | 0 (0%) |
| Dermatitis | 0 (0%) | 0 (0%) | 0 (0%) |
| Late adverse events | Grade 2 | Grade 3 | Grade 4 |
| Mucositis | 0 (0%) | 0 (0%) | 0 (0%) |
| Dermatitis | 0 (0%) | 1 (5%) | 0 (0%) |
| Brain necrosis | 1 (5%) | 0 (0%) | 0 (0%) |
| Peripheral motor neuropathy | 0 (0%) | 1 (5%) | 0 (0%) |
| Pneumonitis | 1 (5%) | 0 (0%) | 0 (0%) |
| Urinary tract obstrucion | 0 (0%) | 3 (14%) | 0 (0%) |
